# Translation, Validation and Cross-Cultural Adaptation of a Simplified-Chinese Version of the Tegner Activity Score in Chinese Patients with Anterior Cruciate Ligament Injury

**DOI:** 10.1371/journal.pone.0155463

**Published:** 2016-05-17

**Authors:** Hongshi Huang, Dongxia Zhang, Yanfang Jiang, Jie Yang, Tao Feng, Xi Gong, Jianquan Wang, Yingfang Ao

**Affiliations:** Institute of Sports Medicine, Peking University Third Hospital, Beijing, China; BG Trauma Center Ludwigshafen, GERMANY

## Abstract

**Aims:**

To translate the English version of Tegner Activity Score into a Simplified-Chinese version (Tegner-C) and evaluate its psychometric properties.

**Methods:**

Tegner-C was cross-culturally adapted according to established guidelines. The validity and reliability of Tegner-C were assessed in 78 participants, with 19–20 participants in each of the four groups: before anterior cruciate ligament reconstruction (pre-ACLR) group, 2–3 months after ACLR group, 3–12 months after ACLR group, and healthy control group. Each participant was asked to complete the Tegner-C and Chinese version of International Knee Documentation Committee Subjective Knee Form (IKDC-SKF-C) twice, with an interval of 5±2 days. Intra-class correlation coefficient (ICC_2, 1_) was used to assess the reliability and Spearman’s rank correlation was used for construct validity.

**Results:**

The ICC_2,1_ was higher than 0.90 for all groups except in the pre-ACLR group, for which the ICC_2,1_ was 0.71 (0.41, 0.87) (All with p<0.001). The absolute reliability as evaluated by the smallest detectable change was 0.43, 2.12, 0.89, and 0.44 for the healthy control group, pre-ACLR group, 2–3 months after ACLR group, and 3–12 months after ACLR group, respectively. Neither a ceiling effect nor a floor effect was observed for any group. Significant difference was observed for both Tegner-C and IKDC-SKF-C scores between the control and the other three groups (all with p<0.001), and between pre-ACLR and the 2–3 months after ACLR group (p<0.001).

**Conclusions:**

Tegner-C demonstrated comparable psychometric properties to the original English version and thus is reliable and valid for Chinese-speaking patients with ACL injury.

## Introduction

Anterior Cruciate Ligament (ACL) rupture is one of the most common injuries in the knee joint with an estimated prevalence of 1/3000 [1] in USA population and 0.47% among Chinese athletes [2]. ACL rupture can significantly limit the function of knee joint and might reduce the activity level and life quality of patients. It is important to well understand the functional level in activities so that surgeons and rehabilitation specialists can make better decisions regarding the injury [3]. Different knee evaluation scores and scales are available, including the International Knee Documentation Committee Subjective Knee Form (IKDC-SKF), Tegner Activity Scale [4]. The Tegner Activity Score, developed by Tegner and Lysholm [5] in 1985, was a single-page list with 11 items to be chosen from and a score of 0–10 to be assigned. Competitive sports, recreational sports, and work were included in the list. Its original target patients were those with ACL injury. Gradually, it was validated to be effective for evaluation of patients with all types of knee ligament injuries, meniscal tears, knee cartilage lesion, and other knee conditions [4,6,7].

For a self-reported health measurement to be used in a different country, cross-cultural adaptation and vigorous validation are considered important to maintain the content validity of the health measurement across different cultures. The IKDC-SKF has been translated into Chinese version (IKDC-SKF-C) and validated by Fu and Chan[8], and now the IKDC-SKF-C is commonly used in the Chinese community to measure the knee function from patients’ perspective. The Tegner Activity Score, a useful instrument to document self-reported activity level, has been translated into different languages[9,10] and culturally validated in German and Iranian population [11,12,13]. However, no validated Chinese version is available. This study aims to culturally translate the Tegner Activity Score into a Simplified-Chinese version, and to determine its psychological properties including reliability, validity, ceiling and floor effects among patients with ACL injury as well as healthy population.

## Methods

### Translation and Cross-Cultural Adaptation Procedures

The development of a Simplified-Chinese version of the Tegner Activity Score was composed of six steps, including duplicate translation, discrepancy elimination, backward translation, expert evaluation, patient testing, and word refinement [14,15]. At the first step, two independent translators who were native Chinese speakers from the linguistics field translated the English version of the Tegner Activity Score into an initial Simplified-Chinese version (Tegner-C-1), and then agreed on a preliminary common forward translation in a meeting with the investigators to produce the second version (Tegner-C-2). Another two independent translators not involved in the initial steps backward translated the Tegner-C-2 back into the English version. The reverse translation process was performed to check for any conceptual discrepancies in the Simplified-Chinese version. Since no discrepancy was found, the Tegner-C-2 was approved and then evaluated by a committee of two senior orthopaedic surgeons and three physical therapists. Since the expert panel expressed no major concerns on the content of the translated version, the Tegner-C-2 was renamed the Tegner-C-PILOT. At the fifth step, 25 ACL-injured subjects were asked to complete the Tegner-C-PILOT to check for any difficult, upsetting or confusing items. In addition, no ceiling and flooring effects were seen in this cohort. And then the final Simplified-Chinese version, Tegner-C (Table 1), was confirmed with some refined words.

**Table 1:** Simplified-Chinese version of Tegner Activity Score (Tegner-C).

Tegner运动水平评分表			
**10**	**竞技体育**	足球 (国家或国际级)	
**9**	**竞技体育**	足球 (国家级以下)	
		冰球 (6人制冰上曲棍球)	
		摔跤	
		体操	
**8**	**竞技体育**	班迪球 (8–11人制冰上曲棍球)	
		壁球或羽毛球	
		田径运动 (跳跃项目等)	
		高山滑雪	
**7**	**竞技体育**	网球	
		田径运动 (跑步项目等)	
		摩托车越野赛、速度赛	
		手球	
		篮球	
	**娱乐体育**	足球	
		冰上曲棍球 (6人制或8–11人制)	
		壁球	
		田径运动 (跳跃项目等)	
		娱乐或竞技性定向越野赛	
**6**	**娱乐体育**	网球和羽毛球	
		手球	
		篮球	
		高山滑雪	
		慢跑 (每周至少五次)	
**5**	**工作**	重体力劳动 (从事建筑业、林业工作等)	
	**竞技体育**	自行车赛	
		越野滑雪	
	**娱乐体育**	不平整路面慢跑 (每周至少两次)	
**4**	**工作**	中等体力劳动 (货车驾驶、重家务劳动等)	
	**娱乐体育**	自行车赛	
		越野滑雪	
		平整路面慢跑 (每周至少两次)	
**3**	**工作**	轻体力劳动 (如护理工作)	
	**娱乐体育**	游泳	
	**可在山林行走**		
**2**	**工作**	轻体力劳动	
	**可在不平整路面行走, 但不能在山林行走**		
**1**	**工作**	坐着工作	
	**可在平整路面行走**		
**0**	**因膝关节问题休病假或领残废补助**		
**评分 (0–10分): ___________**			

### Validation procedure

A sample size of 20 subjects in each subgroup was required to measure a correlation of 0.6 between Tegner-C and IKDC-SKF-C scores, with a power of 0.8 at a significance level of 0.05. Eighty subjects were recruited into this study, with 20 subjects in each of the following four groups: pre-ACLR group (before ACL reconstruction); postsurgical rehabilitation early phase group (2–3 months after ACL reconstruction); postsurgical rehabilitation late phase group (3–12 months after ACL reconstruction); healthy control group. For the former three groups (ACL-injured groups), the inclusion criterion was ACL rupture accompanied with no or minimal injuries to other tissues so that do not need surgical intervention. The exclusion criteria included: history of other injuries affecting the lower extremity or back, systematic inflammatory rheumatic disease, osteoarthritis, neurological or vascular conditions and psychiatric disorders. Diagnosis of ACL injury was made by the orthopedic surgeons based on physical examination and magnetic resonance imaging, and then confirmed by arthroscopy evaluation. Healthy control group included healthy volunteers who had never had knee injuries or pain.

The study protocol was approved by the Institutional Research Board of Peking University Third Hospital ((IRB00006761-2014211) and the written informed consents were obtained from all subjects.

### Instruments

The Tegner Activity Score was developed by Tegner and Lysholm in 1985 [5]. An activity level of 10–6 corresponds to participation in competitive and/or recreational sports, 5–1 corresponds to participation in recreational sports and heavy /moderate/ light labor working, and 0 is recorded for a sick leave or disability pension because of knee problems [5,6].

The Chinese version of International Knee Documentation Committee Subjective Knee Form (IKDC-SKF-C) was shown to be a reliable and valid tool to assess knee function in Chinese population [8]. It consisted of 3 domains and 18 items, covering symptoms, sports and daily activities, current and pre-injury knee functional status. Each item has various response options. The range of score is from 0 to 100.

This validation study was prospective and mono-centered. To ensure that the status remained stable between the repeated measurements and the research was feasible, each participant completed the IKDC-SKF-C and Tegner-C twice with an interval of 5±2 days. During the first measurement, all participants filled out the Tegner-C and IKDC-SKF-C questionnaires in the presence of the investigator; while the second measurements were done via contact by phone. In addition, the subjects were explicitly asked the question: ‘Has your status changed since filling out the initial IKDC-SKF-C and Tegner-C questionnaires?’ The possible responses were: (a) No; (b) Yes, the problem changed for the better or for the worse. Only subjects with no change in their knee functions within the time interval were included in the retest day [11].

### Data analysis

STATA 13.0 (Statacorp, USA) was used for all statistical analyses. The significance level was set at 0.05. Data normality was checked by inspection of histograms and Shapiro–Wilk test. The test-retest reliability was then examined using intra-class correlation coefficient (ICC_2, 1_) with the two-way random effects’ model proposed by Shrout and Fleiss [16]. The standard error of measurement (SEM) and the smallest detectable change (SDC) were calculated to assess the absolute reliability [17,18]. The SDC represents the minimal change that one needs to achieve to ensure that the observed change is true, not measurement error.

Validity refers to how precise the “true value” estimated by the questionnaire is. The content validity was evaluated by the distribution of the Tegner-C score and represented by the floor or ceiling effect. Floor effect was determined as the proportion of patients who obtained the lowest possible score, and ceiling effect was determined as the proportion of patients who obtained the highest possible score. A ceiling effect and floor effect of <20% were considered acceptable. To evaluate the construct validity of Tegner-C compared to similar and dissimilar concepts of the IKDC-SKF-C, the Spearman’s rank correlation between the Tegner-C score and the overall score, as well as the individual score of each question, of the IKDC-SKF-C, was used.

To explore whether Tegner-C was able to discriminate subjects from different groups in the same manner as the IKDC-SKF-C, Kruskal-Wallis one-way analysis of variance and post-hoc Mann-Whitney U tests were performed to compare the medians of Tegner-C and IKDC-SKF-C between the four groups. The significance level for post-hoc analysis was set at 0.05/6 = 0.008. A Bland and Altman plot was added to graphically illustrate the test-retest reliability. For validity assessment, a scatter plot was included to demonstrate the relationship between Tegner-C and IKDC-SKF-C scores.

## Results

### Translation process

During forward and backward translations, no major modifications were made by the translators except that the number of players was specified for “ice hockey” and “bandy” to avoid confusion. No other item was found problematic by the respondents in the adaptation process.

Seventy-eight subjects were included for analysis. Two subjects were excluded because they failed to meet the inclusion criteria. There were 18 females and 60 males, with an average age of 28.92 ± 6.55 years (range: 19–47 years). Three professional athletes were included, with each from the pre-ACLR group, postsurgical rehabilitation early phase group, and healthy control group. More details were described in Table 2.

**Table 2:** The participant characteristics for different groups (N = 78).

	Control Group (n = 20)	ACL-patient^δ^ (n = 58)	Pre-ACLR (n = 20)	2-3months after ACLR (n = 19)	3-12months after ACLR (n = 19)
**Age, years, mean (SD)**	27.25 (6.61)	29.50 (6.46)	28.40 (3.38)	30.16 (7.81)	30.00 (7.39)
**Sex, Female/Male**	14/6	4/54	0/20	3/16	1/18
IKDC-SKF-C, mean (SD)*	98.07 (2.67)	67.70 (17.76)	74.34 (13.67)	54.33 (17.59)	74.08 (14.15)
Tegner-C, mean (SD)*	5.33 (0.92)	3.68 (1.60)	4.10 (1.43)	2.63 (1.42)	4.29 (1.43)

ACLR: anterior cruciate ligament reconstruction; SD: Standard deviation; IKDC-SKF-C: International Knee Documentation Committee Subjective Knee Form Chinese version; Tegner-C: Tegner Activity Scale Simplified-Chinese version

^**δ**^ All patients with ACL-patient (pre-ACLR, 2–3 months after ACLR or 3–12 months after ACLR)

* IKDC-SKF-C and Tegner-C scores were calculated as the average of two measurements

### Reliability

**Fig 1:**
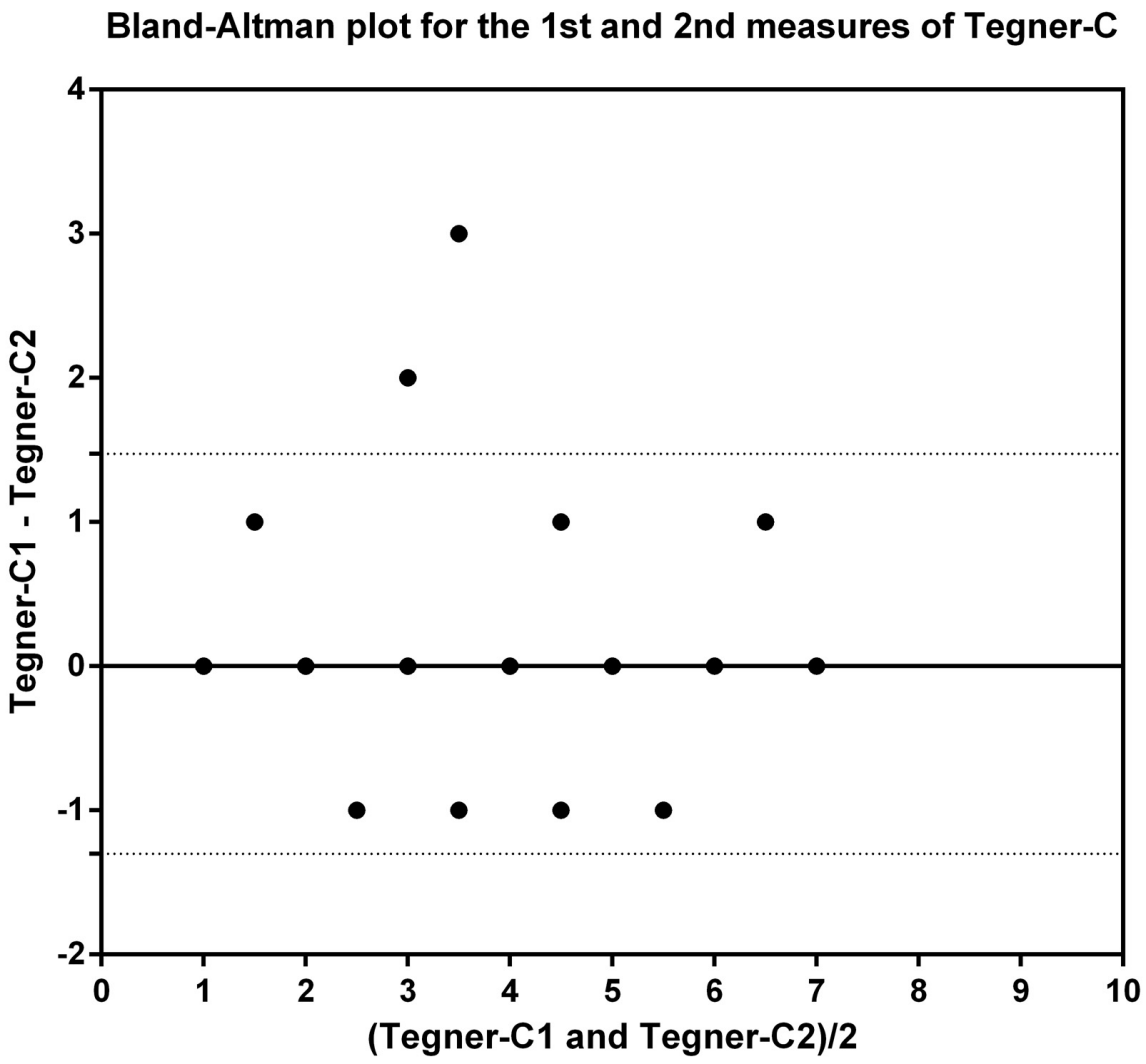
A Bland and Altman plot for test-retest reliability of the Tegner-C (only for ACL-patients).

The ICC_2,1_ was 0.97 (0.93, 0.99) for healthy controls, 0.90 (0.84, 0.94) for the ACL-patient group (including three groups: pre-ACLR, 2–3 months after ACLR or 3–12 months after ACLR), 0.71 (0.41, 0.87) for pre-ACL group, 0.95 (0.87, 0.98) for 2–3 months after ACLR group, and 0.99 (0.97, 1.00) for 3–12 months after ACLR group. The SEM was 0.16, 0.50, 0.77, 0.32, and 0.16 for the healthy controls, ACL-patient group, pre-ACL-reconstruction group, 2–3 months after ACLR group, and 3–12 months after ACLR group, respectively, with the resulting SDC of 0.43, 1.38, 2.12, 0.89, and 0.44 respectively (Table 3). Differences between the first and second measurement seemed to have constant variance as shown in the Bland-Altman plot (Fig 1).

**Table 3:** The test-retest reliability parameters of the Tegner-C scores in different groups.

	1^st^ measurement mean (SD)	2^nd^ measurement mean (SD)	Spearman Rho	ICC (95% CI)	SEM	SDC
**Control Group**	5.30 (0.92)	5.35 (0.93)	0.96	0.97 (0.93, 0.99)	0.16	0.43
**ACL-patient group**	3.72 (1.55)	3.64 (1.65)	0.90	0.90 (0.84, 0.94)	0.50	1.38
**Pre-ACLR**	4.25 (1.16)	3.95 (1.67)	0.77	0.71 (0.41, 0.87)	0.77	2.12
**2-3months after ACLR**	2.63 (1.46)	2.63 (1.42)	0.95	0.95 (0.87, 0.98)	0.32	0.89
**3-12months after ACLR**	4.26 (1.48)	4.32 (1.42)	0.99	0.99 (0.97, 1.00)	0.16	0.44

ACLR: anterior cruciate ligament reconstruction; SD: Standard deviation; IQR: Inter-quartile range; ICC: Intra-class correlation; SEM: standard error of measurement; SDC: smallest detectable change

### Validity

**Fig 2:**
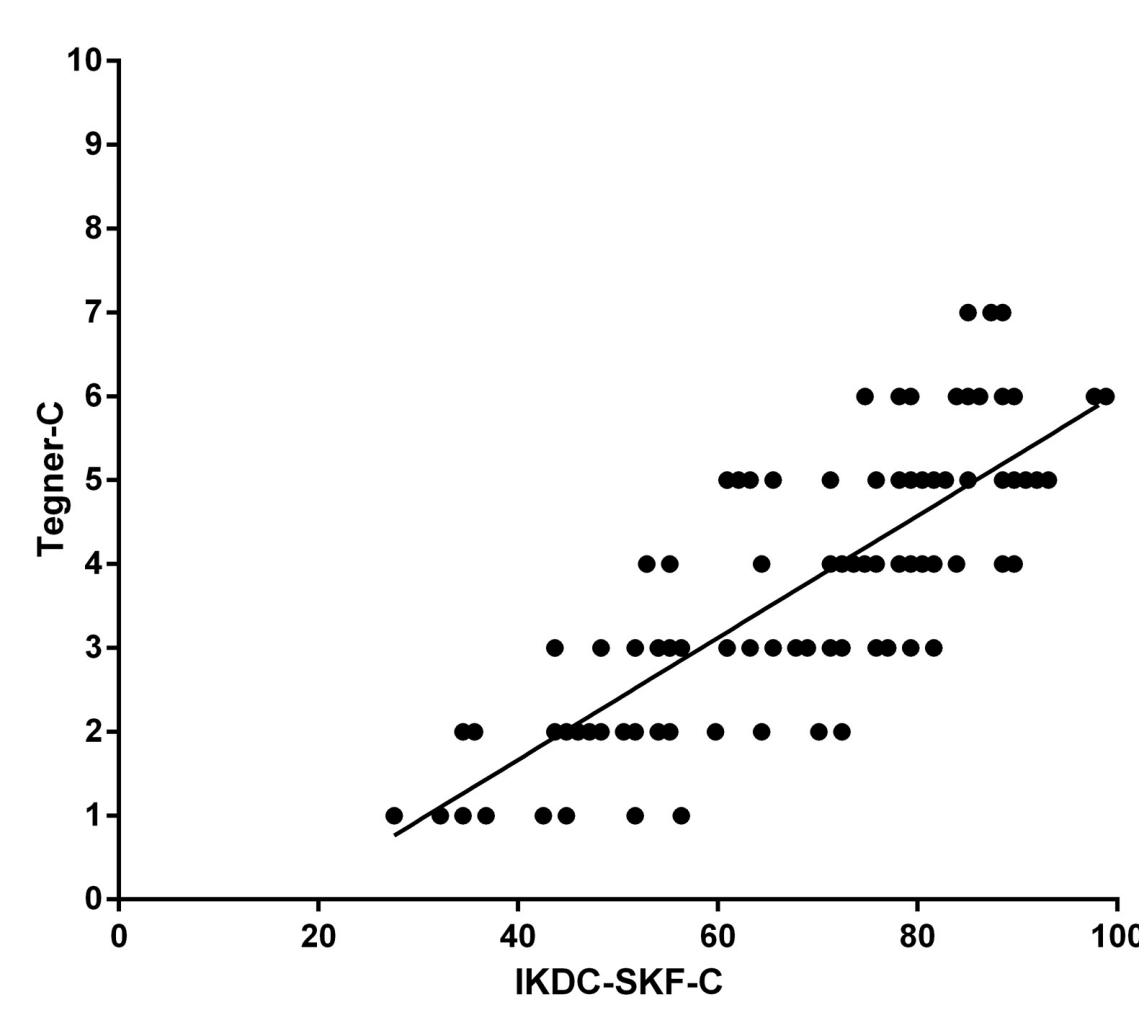
Relationship between Tegner–C and IKDC-SKF-C (only for ACL-patients).

No ceiling or floor effect was observed in either group, with no patients scored the highest (10) or lowest (0) value. As shown in Table 4 and Fig 2, the Tegner-C showed excellent correlation with the IKDC-SKF-C overall score (r = 0.79; p<0.05) and good correlation with the individual scores of questions 1, 5, 7 and 8 (Q_1, 5, 7, 8_), whereas it showed poor correlation with Q_6, 10_ of the IKDC-SKF-C (Table 4).

**Table 4:** Spearman rank correlation coefficients of the Tegner-C score and the IKDC-SKF-C scores in different patient groups.

	Control Group (n = 20)	ACL-patient^δ^ (n = 58)	Pre-ACLR (n = 20)	2–3 months after ACLR (n = 19)	3–12 months after ACLR (n = 19)
**IKDC_overall score**	0.56*	0.84*	0.66*	0.81*	0.66*
**IKDC_Q1 score**	0.60*	0.74*	0.81*	0.59*	0.64*
**IKDC_Q2 score**	-0.20	0.44*	0.29	0.50*	0.31*
**IKDC_Q3 score**	-0.20	0.40*	0.20	0.67*	0.09
**IKDC_Q4 score**	-0.08	0.56*	0.05	0.36*	0.44*
**IKDC_Q5 score**	0.45*	0.72*	0.74*	0.59*	0.58*
**IKDC_Q6 score**	NA	0.19	0.17	0.53*	0.07
**IKDC_Q7 score**	0.51*	0.80*	0.56*	0.68*	0.73*
**IKDC_Q8 score**	0.46*	0.77*	0.67*	0.50*	0.75*
**IKDC_Q9 score** ^§^	0.33*	0.81*	0.58*	0.76*	0.65*
**IKDC_Q10 score**	0.33*	0.06	-0.11	-0.01	0.21

IKDC-SKF-C: Chinese version of International Knee Documentation Committee Subjective Knee Form; Q: Question; ACLR: anterior cruciate ligament reconstruction

^**δ**^ All patients with ACL-patient (pre-ACLR, 2–3 months after ACLR or 3–12 months after ACLR)

*correlation is significant at the 0.05 level (2-tailed)

^§^ The total score of each sub-question in Question 9; NA: In the control group, all participants scored 2 in Question 6

**Fig 3:**
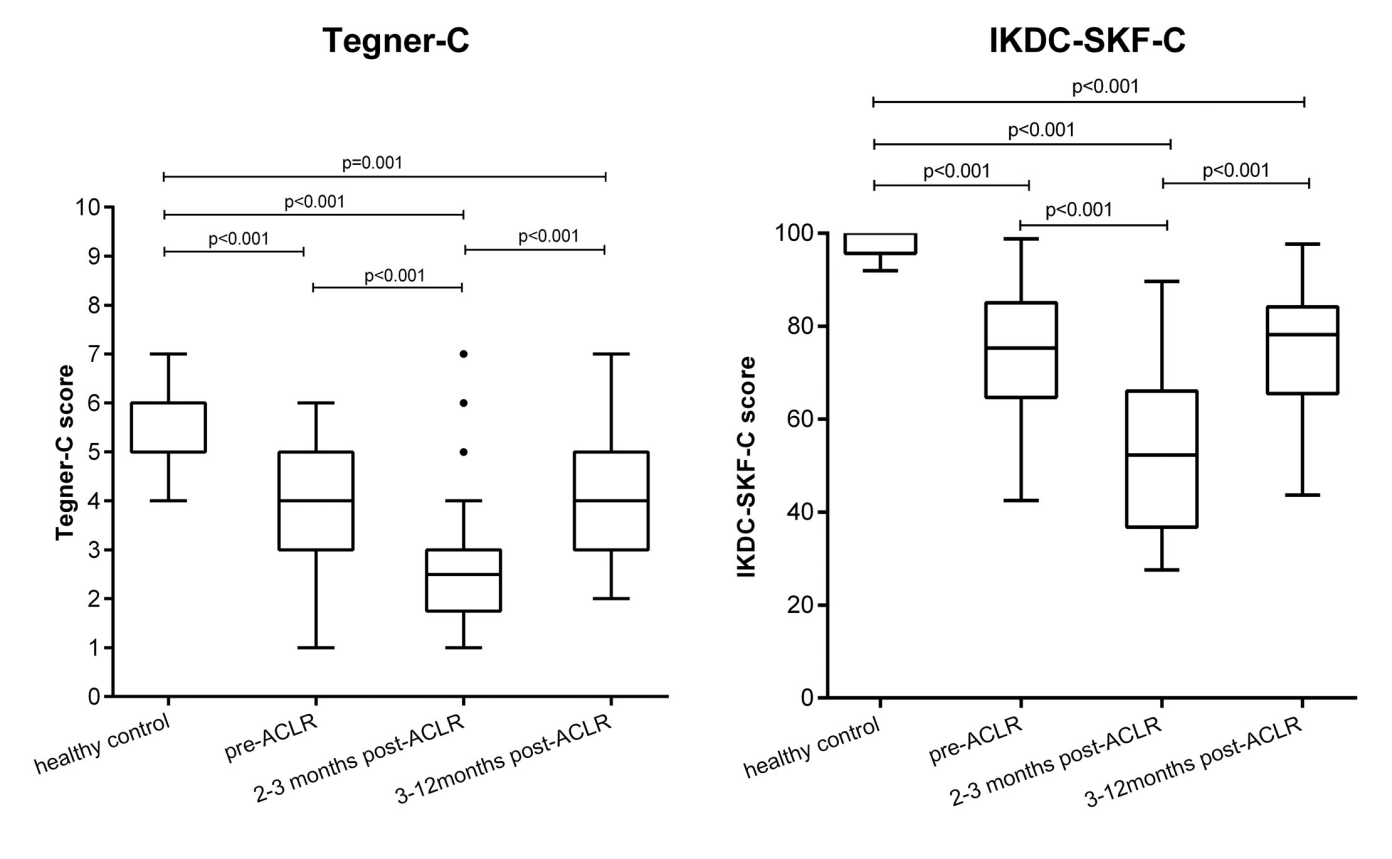
Comparisons of Tegner-C and IKDC-SKF-C among different groups. The Tegner-C and IKDC-SKF-C scores were calculated as the average of two measurements; p-value was obtained from Mann-Whitney U test.

The Tegner-C score was 5.33 ±0.92, 4.10 ±1.43, 2.63 ± 1.42, and 4.29 ± 1.43 in the control group, pre-ACLR group, 2–3 months after ACLR group, and 3–12 months after ACLR group, respectively (Fig 3). The IKDC-SKF-C score was 98.07 ±2.67, 74.34 ±13.67, 54.33 ± 17.59, and 74.08 ±14.15 in the control group, pre-ACLR, 2–3 months after ACLR group, and 3–12 months after ACLR group, respectively (Fig 3). Significant differences were observed for both the Tegner-C and IKDC-SKF-C scores between the control and the other three groups (all with p<0.001), pre-ACLR group and 2–3 months after ACLR group (p<0.001), 2–3 months after ACLR group and 3–12 months after ACLR group (p<0.001) (Fig 3).

## Discussion

This study is the first validated Tegner Activity Score for Chinese patient-administered instruments in an ACL injury population to quantify functional level in daily living and sports activities. The results of this validation study showed that the Tegner-C had acceptable properties in terms of test-retest reliability, content and construct validity and could be used to evaluate the activity level of Chinese patients with ACL injury before and after ACL reconstruction.

The relative reliability of Tegner-C was high. The ICC values were in accordance with the reported values in the literature for the English [4] or Persian version [11] (0.82–0.92) except for the one in the pre-ACLR group, which was only 0.71 (0.41, 0.87) in this study. For the relatively low test-retest reliability observed in pre-ACLR group, it is not likely to be caused by the improvement or reduction in activity level during the intervals between the first and second measurements. The knee function scores as evaluated by the IKDC-SKF-C at the two measurements were almost the same. Several subjects scored 7 at the first measurement but 3 or 4 at the second measurement were observed in the pre-ACL group. It is possible that at the first measurement they might have misunderstood the question as their normal activity level before ACL injury instead of their current activity level. And since there was no introductory text in the original English version, the question was not asked with a specific time window. A short introduction text to inform the patients of the specific time period when the question is addressed to should be created, for example, “during the past four weeks or after ACL injury/ reconstruction”.

The SDC is the smallest value that may be considered as an actual change instead of a measurement error in clinical practice. An SDC of 0.43–0.89 for the Tegner-C (Table 3) was observed in this study except for those before the ACL Reconstruction (SDC = 2.12) that might be due to low test-retest reliability. In the studies by Briggs et al. [6,19] using the original English version, the SEM for patients with ACL injury was 0.64 and 0.4 for patients with meniscus injury, with a resulting SDC of 1.77 or 1.11 when applied to the formula of Beaton, which was used in this study. To ensure that a measured change is not due to measurement error, therefore, a change of 1 point in the Tegner-C must be required in clinical practice.

With regard to construct validity, excellent correlation was seen between the Tegner-C and IKDC-SKF-C [8] overall score (r = 0.79; p<0.05) and good correlation with the Q_1, 5, 7, 8_ of the IKDC-SKF-C, whereas it showed poor correlation with Q_6, 10_ of the IKDC-SKF-C (r = 0.00–0.53). This may imply that Tegner-C scale has higher correlations with those subscales of IKDC that measure pain or level of activity than with those subscales that measure stiffness, swelling, locking or catching. Briggs et al also reported that patients with more difficulty in activity of daily life and sports activities had lower Tegner score (p<0.05) [19]. The poor correlation between Tegner-C and Question 10 of IKDC-SKF-C which asks about the general knee function among patients with ACL injury or after ACL reconstruction might indicate the importance of recording both the activity level and knee function in clinical practice. It has been hypothesized that instruments with acceptable content validity would have fewer ceiling and floor effects. In our study no ceiling and floor effects were seen for the Tegner-C, which was also reported in validity study of Tegner in patients with patellar dislocation [20], while acceptable ceiling effects of 3% and floor effects of 8% were seen in the study of ACL-injured patients [19]. In addition, ceiling and floor effects of 2.5% were reported in the meniscal-injured populations [6].

In this study, subjects were divided into different groups: healthy controls, ACL patients before and after reconstruction. In addition, the postoperative patients were divided into early rehabilitation phase (2–3 months) and late rehabilitation phase (3–12 months) groups. Both the IKDC-SKF-C and Tegner-C were able to discriminate patients (before or after ACLR) from healthy controls, early rehabilitation phase group from pre-ACLR group, and late rehabilitation phase group from early rehabilitation phase group. This seems to indicate that Tegner-C has similar value as IKDC-SKF-C in terms of discriminating different population groups.

Limitations of the study include that the sensitivity of the scale to document changes (responsiveness) was not measured, which would be necessary for full coverage of the psychometric properties of the Tegner-C. However, the scores between different patient groups were compared. And the results showed that Tegner-C was able to discriminate between patients in the same manner as IKDC-SKF-C. The patient in this study was limited to isolated ACL injuries and a significant male proportion ((54/58)) of ACL patients, which might limit the generalizability of the results. In addition, the criterion validity was not determined in this study since there is no gold standard among the knee scores for the Tegner activity scale and itself was recommended as the gold standard.

In conclusion, the Tegner-C is a reliable and valid instrument to evaluate activity level of patients with ACL injuries in Chinese population. Future studies are needed to investigate the properties of Tegner-C for Chinese patients with other knee problems such as meniscal injury, patellar dislocation and knee arthroplasty.

## Supporting Information

S1 Dataset The detail information of the subjects included for analysis. (XLS)
